# RandseqR: An R Package for Describing Performance on the Random Number Generation Task

**DOI:** 10.3389/fpsyg.2021.629012

**Published:** 2021-05-04

**Authors:** Wouter Oomens, Joseph H. R. Maes, Fred Hasselman, Jos I. M. Egger

**Affiliations:** ^1^Center of Excellence for Neuropsychiatry, Vincent van Gogh Institute for Psychiatry, Venray, Netherlands; ^2^Donders Institute for Brain, Cognition and Behavior, Radboud University, Nijmegen, Netherlands; ^3^School for Pedagogical and Educational Science, Radboud University, Nijmegen, Netherlands; ^4^Stevig, Specialized and Forensic Care for People With Intellectual Disabilities, Dichterbij, Oostrum, Netherlands

**Keywords:** recurrence qualification analysis, R-package, executive function, contextual neuropsychology, random number generation, assessment

## Abstract

The Random Number Generation (RNG) task has a long history in neuropsychology as an assessment procedure for executive functioning. In recent years, understanding of human (executive) behavior has gradually changed from reflecting a static to a dynamic process and this shift in thinking about behavior gives a new angle to interpret test results. However, this shift also asks for different methods to process random number sequences. The RNG task is suited for applying non-linear methods needed to uncover the underlying dynamics of random number generation. In the current article we present RandseqR: an R-package that combines the calculation of classic randomization measures and Recurrence Quantification Analysis. RandseqR is an easy to use, flexible and fast way to process random number sequences and readies the RNG task for current scientific and clinical use.

## Introduction

The Random Number Generation (RNG) task has potential as an easy to administer and concise assessment tool of executive functioning (EF). The rationale behind the RNG paradigm is simple: it requires executive control (i.e., inhibition of prepotent responses and monitoring of working memory content) to avoid deterministic (i.e., non-random) behavior. Over the years, several measures have been proposed that quantify RNG performance based on deviations from mathematical randomness. Research has shown that these randomization measures could be attributed to different aspects of EF, namely inhibition of prepotent responses and updating of working memory (Towse and Neil, 1998; Miyake et al., 2000; Peters et al., 2007; Maes et al., 2011). It is noteworthy that these aspects of EF imply a strong dependence on the temporal structure of the response sequence. Both inhibition of number selection and updating of working memory content is a function of previously selected numbers. However, randomization measures only explain this temporal structure on minimum timescales. For example, **redundancy** expresses the inequality of response usage (Shannon, 1948), while **RNG** expresses the difference between the observed and mathematical diagram distribution (Evans, 1978).

In general, randomization measures are not sensitive to a disruption of the temporal structure of a sequence. In a large pool of experimental and simulated time-series (including random sequences), Giuliani et al. (2001), distinguished between the information gained from an order-dependent analysis and an order-independent analysis of time series and clearly emphasized the role of information as an order-dependent process. This clearly shows that the temporal structure of the response sequence contains a wealth of information about the underlying executive behavior and corresponds to the notion that variability in behavioral data is not mere random fluctuation (Gilden, 2001; Van Orden et al., 2003). To fully understand executive behavior, it is paramount to use complexity methods that quantify the characteristics of any temporal pattern (Shockley, 2005; Webber and Zbilut, 2005). Recurrence quantification analysis (RQA) is such a complexity method, which is applicable to categorical (Dale and Spivey, 2005; Dale et al., 2011) and relatively short time series, like random number sequences.

Quantifying the performance on the RNG task through calculating randomization measures is an onerous task. Two decades ago, Towse and Neil (1998) developed software (i.e., RGcalc) to make the computation of randomization measures more manageable and accessible. Nowadays, the functionality of RGcalc is increasingly obsolescent and it offers little flexibility. Although RQA is a relatively new method, there are already several toolboxes available to compute RQA measures. The first of these toolboxes (The Cross Recurrence Plot toolbox for MATLAB) was released in 2007 by Marwan and in 2014 Coco and Dale released an R package to perform RQA. More recently, Hasselman (2017) developed an R package for studying Complex Adaptive System and NETworks (*casnet*), that includes extensive RQA functionality. To make the RNG task accessible for current scientific and clinical use we compiled both methods (randomization measures and RQA measures) into a single R package (*RandseqR*). RandseqR encompasses functions to compute randomization measures, based on mathematical equations taken from Towse and Neil (1998) and RQA functionality imported from *casnet*. We explain the functionality of *RandseqR* in the present paper.

### RandseqR

For the reproduction of the randomization measures, the source code of the *RGcalc* software was made available to us by John Towse. The classic randomization measures available in *RandseqR* are Redundancy (R), RNG, RNG2, Coupon, Null-Score Quotient (NSQ), First-Order Difference (FOD), Adjacency, Turning Point Index (TPI), Phase Length, Runs, Repetition Distance, Repetition Gap, and Phi index. For a full explanation of these measures, see Towse and Neil (1998).

To smooth out the output in *RandseqR* (compared to RGcalc), small alterations are made to the default calculation of the randomization measures. First, *RGcalc* returns percentages for some randomization measures, while returning proportions (i.e., a value between 0 and 1) for other measures. For example, *RGcalc* returns percentages for **R**, while returning proportions for **RNG** (which is a redundancy measure for diagrams instead of single numbers). For these measures *RandseqR* returns proportions of 1. For **TPI**, which can have values >100%, *RandseqR* returns a value with a mean of 0 (ranging from −1 to 1), similar to the output of **phi-index**, which has a mean 0 and values ranging from -infinite to infinite. The output was left the same for all measures where the above is not applicable, like **coupon** and the frequency distributions. Secondly, *RGcalc* pairs the last digit in the number sequence to the first for some randomization measures (**RNG**), but not for all measures (for **RNG2** *RGcalc* does not do this). Since the diagrams created by this pairing have no relevance toward the executive construct under study (e.g., inhibition of prepotent responses), *RandseqR* never pairs the end of the number sequence to the start. Lastly, according to Towse and Neil (1998), **Runs** (the variability of **Phase lengths**) is only calculated over ascending **Phase Lengths** in *RGcalc*, whereas *RandseqR* calculates **Runs** over both ascending and descending **Phase Lengths**. Alternatively, *RandseqR* has the option to override the default calculation of the randomization measures in order to replicate output similar to *RGcalc*, with the exception of **Runs**, which could not be reproduced even with the *RGcalc* source code.

A quantitative description of recurrence is given by the following RQA measures: Recurrence Rate (RR), Determinism (DET), Laminarity (LAM), maximal diagonal line length (Lmax), mean diagonal line length (Lmean), entropy of diagonal line length distribution (Lentr), maximal vertical line length (Vmax), Trapping Time (TT), and entropy of vertical line length distribution (Ventr). For a full explanation of these RQA measures, see Marwan et al. (2007) and Hasselman (2017).

For a tutorial on RQA in R, see Wallot (2017). Although the packages/functions used in this paper are different from those implemented in *RandseqR*, basic considerations and recommendations are independent of the choice of package. Of importance is that RQA measures are affected by the following parameters: embedding dimension (*M*), time delay (τ), minimal line length, and radius. To optimize the information for categorical and discontinuous time series (like nominal number sequences), the default value for both (*M*) and (τ) is set to 1 (Dale and Spivey, 2005; Webber and Zbilut, 2005; Dale et al., 2011; Coco and Dale, 2014). The minimal line length is set to 2 to ensure that every recurring combination of two or more digits is considered a diagonal or vertical line structure. The radius is set to <1, such that only exact matches are considered recurrent (Orsucci et al., 1997). See Supplementary Material for availability of *RandseqR*.

### Randomization Measures

### Redundancy

**R** is a measure for inequality of response usage. An R score of 0 equals minimum redundancy (i.e., each response alternative is given in equal proportion) and an R score of 1 equals maximum redundancy (i.e., the same response is given throughout). R is computed as:

$$R=1-\frac{log_{2}(n)-\frac{1}{n}\left(\sum a_{i}*log_{2}(a_{i})\right)}{log_{2}(a)},$$

where *n* is the length of the number sequences, *a* is the total number of response alternatives, and *a*_*i*_ is the number of occurrences of the *i*th response alternative. The numerator equals the amount of observed redundancy in the sequence and the denominator equals the amount of maximum redundancy.

### RNG, NSQ, and RNG2

Like redundancy, **RNG** is a measure of inequality of response usage at the level of diagrams at time lag 1 (i.e., adjacent responses). RNG is computed as:

$$RNG=\frac{\sum n_{ij}*log_{2}\left(n_{ij}\right)}{\sum a_{i}*log_{2}\left(a_{i}\right)},$$

where *n*_*ij*_ is the frequency count of all observed diagrams and *a*_*i*_ is the number of occurrences of the *i*th response alternative.

**NSQ** is a measure for diagrams at time lag 1 that do not appear in the number sequence. NSQ is the opposite of RNG and NSQ is computed as:

$$NSQ=\frac{NS}{a^{2}-1},$$

where *NS* is the diagram alternatives that do not appear in the number sequence and *a* is as above.

**RNG2** is a measure of inequality of diagram usage at time lag 2 (i.e., interleaved responses). The computation is similar to that of the RNG measure, where *n*_*ij*_ is the frequency count of all observed diagrams at time lag 2.

### Coupon

**Coupon** is a measure of the cycling pace of response alternatives, expressed as the mean number of responses before all response alternatives are used. When one or more response alternatives are not used at all, a coupon score cannot be calculated.

### First-Order Difference

**First-order differences** are presented as a frequency table of the arithmetic differences between numbers at time lag 1 (i.e., the difference between the *N*_*i*_ response and the *N*_*i*−1_ response).

### Adjacency

**Adjacency** is the number of diagrams at time lag 1 with an ordinal sequence of response alternatives. Ascending diagrams have a *first-order difference* of −1 and descending diagrams have a *first-order difference* of +1. Adjacency is computed as:

$$A=\frac{number\;of\;adjacent\;pairs}{total\;number\;of\;response\;pairs},$$

and adjacency is calculated for both ascending and descending diagrams as well as a total adjacency score.

### Turning Point Index, Phase Length, and Runs

**TPI** is a measure of ascending and descending flow in the number sequence. TPI is calculated by counting the number of points in the sequence that mark a change in numerical direction (from ascending to descending or vice versa) and comparing this to the expected value of numerical changes in a random sequence. TPI is computed as:

$$TPI=\left(\frac{TP_{observed}}{\frac{2}{3}*\left(n-2\right)}\right)-1.$$

The denominator is the expected amount of turning points in a sequence of length *n*. A TPI of >0 equals more numerical changes than expected, relative to a random sequence.

The intervals between turning points are called **Phase lengths**. Both ascending and descending phase lengths are presented as a frequency table, while **Runs** is the variance in phase lengths. In *RGcalc*, runs is the variance of ascending phase lengths only.

### Repetition Distance, Repetition Gap, and Phi Index

**Repetition distance** is the distance or gap between number repeats in the sequence, presented as a frequency table. From this frequency table three **repetition gap** scores are derived: the **mean gap**, the **median gap**, and the **modal gap**. The **Phi Indices** are somewhat related to repetition distance because these are the ratio between the observed repetition distance at time lag *x* and the expected distance at time lag *x*. However, the calculation of phi is complex and contra-intuitive (Wagenaar, 1970; Wiegersma, 1984; Towse and Neil, 1998; see Supplementary Materials for an extensive account on the calculation of the Phi indices).

### RQA Measures

**Figure 1:**
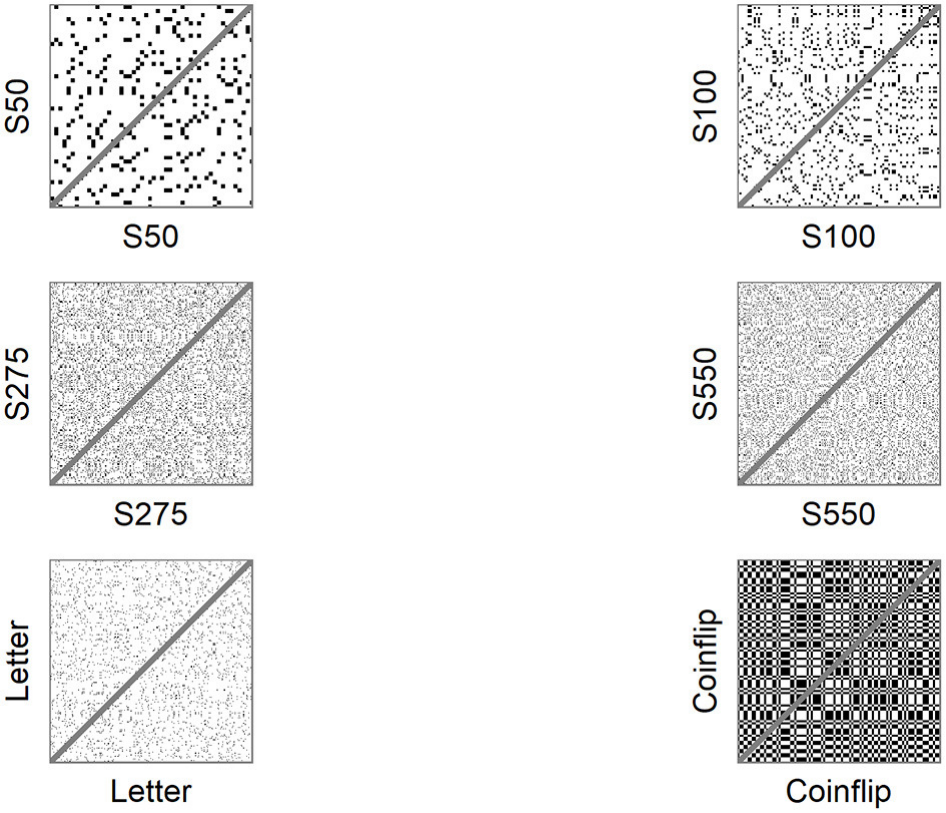
Auto-recurrence plots of sample sequences.

To explain the concept of recurrence, consider an auto-recurrence plot (auto-RP). An auto-RP is created by plotting a number sequence *x* of length *N* on both axes in an *N x N* matrix and marking every point at (*i, j*), whenever *x*(*j*) has the same number as *x*(*i*). Due to the auto-recurrent nature, these RPs are symmetrical regarding the diagonal *i = j* and both planes of the RP contain the same information (see **Figure 1**). **RR** is the proportion of recurring numbers (black dots) to non-recurring numbers (white dots), ignoring the main diagonal. A number in the time series is considered recurring if it falls within a given radius (for nominal number sequences this means that only matching numbers are considered recurring). If two or more recurring numbers are adjacent, they form a line structure. **DET** is the proportion of recurring numbers that form diagonal line structures, while **LAM** is the proportion of recurring numbers that make up the vertical (or horizontal) line structures. The complexity of these line structures is summarized by the average line length, the longest line length, and the variance in line length distribution; a high variance equals a higher uncertainty of a line of given length occurring (entropy).

### Recurrence Rate

**RR** is the proportion of recurrence in the number sequence (i.e., the proportion of marked points to non-marked points in the auto-RP). RR is computed as:

$$RR=\frac{1}{N^{2}}\overset{N}{\underset{i,j=1}{\sum }}R_{i,j},$$

where *N* is the length of the number sequence.

### Diagonal Line Structures

**DET** is the proportion of recurring points forming diagonal line structures and quantifies the number of repetitive patterns. Based on the default parameter settings, every repetition of two or more consecutive numbers is considered a line structure. DET is computed as:

$$DET=\frac{\overset{N}{\underset{l=l_{min}}{\sum }}lP\left(l\right)}{\overset{N}{\underset{l=1}{\sum }}lP\left(l\right)},$$

where *P*(*l*) is the histogram of the lengths *l* of the diagonal lines.**Lmax** and **Lmean** are measures of stability of these diagonal patterns. Lmax is computed as:

$$L_{max}=max\left(l_{i};i=1,...,N_{l}\right),$$

where *N*_*l*_ is the amount of diagonal line structures in the recurrence plot, and Lmean is calculated as:

$$L_{mean}=\frac{\overset{N}{\underset{l=l_{min}}{\sum }}lP\left(l\right)}{\overset{N}{\underset{l=l_{min}}{\sum }}P\left(l\right)}.$$

**Lentr** is the Shannon information entropy of the probability distribution of the diagonal line lengths *p*(*l*) and is indicative of the complexity of the deterministic structures of the number sequence. Lentr is calculated as:

$$Lentr=-\overset{N}{\underset{l=l_{min}}{\sum }}p\left(l\right)\ln*p\left(l\right).$$

### Vertical Line Structures

**LAM** is the proportion of recurring points forming vertical line structures and quantifies the amount of repeating numbers. Based on the default parameter settings, every repetition of two or more of the same number is considered a vertical line structure. LAM is calculated as:

$$LAM=\frac{\overset{N}{\underset{v=v_{min}}{\sum }}vP\left(v\right)}{\overset{N}{\underset{v=1}{\sum }}vP\left(v\right)},$$

where *P*(*v*) is the histogram of the lengths *v* of the diagonal lines.**Vmax** and **TT** are measures of stability of these vertical line structures. Vmax is computed as:

$$V_{max}=max\left(v_{i};i=1,...,N_{v}\right),$$

where *N*_*v*_ is the number of vertical line structures in the recurrence plot, and TT is calculated as:

$$TT=\frac{\overset{N}{\underset{v=v_{min}}{\sum }}vP\left(v\right)}{\overset{N}{\underset{v=v_{min}}{\sum }}P\left(v\right)}.$$

**Ventr** is the Shannon information entropy of the probability distribution of the vertical line lengths *p*(*v*) and is indicative of the complexity of *trapped states* of the number sequence. Ventr is calculated as:

$$Ventr=-\overset{N}{\underset{v=v_{min}}{\sum }}p\left(v\right)\ln*p\left(v\right).$$

### Example

In the following section, we illustrate the randseqR package based on several computer-generated random sequences: random number sequences from 1 to 9 with a length of respectively 50, 100, 275, and 550 numbers, a sequence of 275 random letters and a sequence of 100 coinflips (the sequences were all generated using the base R *sample* function with the seed set to 42). The main functions in the *RandseqR* package are *allRNG*, which calculates all randomization measures, and *crqa_cl*, which is imported from casnet and calculates the RQA measures. For the use of the latter we refer to the extensive casnet documentation (Hasselman, 2017). The use of *allRNG* is quite straightforward and is of the following form:

$$\begin{array}{l} allRNG\left(y,\;minScale,\;maxScale,\;responseAlternatives,\;results,\;\ldots \right), \end{array}$$

where *y* is the sequence for which to calculate the randomization measures. *minScale* and *maxScale* are the minimum and maximum value, respectively in the observed sequences. Based on this minimum and maximum value *RandseqR* calculates all possible response alternatives. Alternatively, it is possible to define all possible response alternatives with *responseAlternatives*. In this case *minScale* and *maxScale* are derived from this set of response alternatives. The term *responseAlternatives* is used for non-numeric sequences, like random letters or random months, or for number sequences that do not allow for some responses, like only even numbers. The term *results* controls the output, either *classical* (similar to *RGcalc*) or *RandseqR*, and is defaulted to *RandseqR*. Optionally, it is possible to disable the calculation of one or more randomization measures. This is controlled by the output terms: *Redundancy, RNG, RNG2, RF, Coupon, NSQ, FOD, Adjacency, TPI, PhL, Runs, repDistance, repGap*, and *PhiIndex*. By default *allRNG* calculates all randomization measures. In addition to the *allRNG* function, randseqR supports functions for the calculation of separate measures. These work the same as *allRNG* and can be thought of as *allRNG* with all the other output measures disabled. For example:

coupon(y,minScale,maxScale,responseAlternatives,results).

The randomization functions in randseqR have two mandatory terms: *y* and one of *minScale* and *maxScale* or *responseAlternatives*. *Results* and the output terms have default settings and are, therefore, optional. The following code was used to calculate part of the output in Table 1:

$$allRNG\left(x,\;minScale=1,\;maxScale=9,\;results=\;“randseqR^{\prime \prime }\right),$$

where *x* is a sequence of random numbers of length *n*,

$$allRNG\left(y,\;responseAlternatives=letters,\;results=\;“classical^{\prime \prime }\right),$$

where *y* is a sequence of random letters of length 275 and, *letters* contain all 26 letters of the alphabet, and

$$\begin{array}{l} allRNG(z,\;responseAlternatives=c\left(“head{,}^{\prime \prime }“tail^{\prime \prime }\right),\;results\\ \;=“randseqR^{\prime \prime }), \end{array}$$

were *z* is a sequence of random coinflips of length 100. These three lines of code calculates the *RandseqR* output for the number sequences, the *classical* output for the letter sequence, and the *RandseqR* output for the coinflip sequence, respectively as shown in Table 1.

**Table 1:** RNG output for RGcalc, RandseqR (classical), and RandseqR (default).

**Sequence**	**RGcalc**	**Classical**	**randseqR**
**Redundancy**			
S50	3.289	3.289	0.033
S100	2.402	2.403	0.024
S275	0.374	0.374	0.004
S550	0.453	0.453	0.005
Letters	1.565	1.565	0.016
Coinflip	0.116	0.115	0.001
**RNG**			
S50	0.223	0.223	0.208
S100	0.295	0.295	0.286
S275	0.396	0.396	0.393
S550	0.486	0.486	0.485
Letters	0.117	0.187	0.183
Coinflip	0.823	0.823	0.812
**NSQ**			
S50	55.000	55.000	0.550
S100	35.000	35.000	0.350
S275	1.250	1.250	0.012
S550	0.000	0.000	0.000
Letters	67.407	68.593	0.686
Coinflip	0.000	0.000	0.000
**RNG2**			
S50	0.176	0.177	0.177
S100	0.279	0.279	0.279
S275	0.387	0.387	0.387
S550	0.489	0.489	0.489
Letters	0.116	0.178	0.178
Coinflip	0.802	0.802	0.802
**TPI**			
S50	103.125	103.125	0.031
S100	96.429	96.429	−0.036
S275	91.758	91.758	−0.082
S550	93.339	93.339	−0.067
Letters	96.154	96.154	−0.038
Coinflip	78.061	78.061	−0.219
**Runs**			
S50	0.129	NA	0.451
S100	0.288	NA	0.906
S275	0.739	NA	0.781
S550	0.845	NA	0.770
Letters	0.993	NA	0.655
Coinflip	0.000	NA	1.210
**Coupon**			
S50	27.000	27.000	27.000
S100	22.500	22.500	22.500
S275	25.100	25.100	25.100
S550	25.950	25.952	25.952
Letters	91.330	91.333	91.333
Coinflip	2.850	2.853	2.853
**Ascending**			
S50	6.000	6.000	0.060
S100	8.000	8.000	0.070
S275	10.180	10.182	0.102
S550	8.910	8.909	0.089
Letters	6.180	6.182	0.062
Coinflip	26.000	26.000	0.260
**Descending**			
S50	16.000	16.000	0.160
S100	7.000	7.000	0.070
S275	8.730	8.727	0.087
S550	11.820	11.818	0.118
Letters	4.730	4.727	0.047
Coinflip	26.000	26.000	0.260
**Combined**			
S50	22.000	22.000	0.220
S100	15.000	15.000	0.140
S275	18.910	18.909	0.189
S550	20.730	20.727	0.207
Letters	10.910	10.909	0.109
Coinflip	52.000	52.000	0.520
**RG mean**			
S50	7.320	7.317	7.317
S100	7.990	7.989	7.989
S275	8.740	8.744	8.744
S550	8.930	8.928	8.928
Letters	23.060	23.056	23.056
Coinflip	1.980	1.980	1.980
**RG median**			
S50	6.000	6.000	6.000
S100	5.000	5.000	5.000
S275	6.000	6.000	6.000
S550	7.000	7.000	7.000
Letters	15.000	15.000	15.000
Coinflip	2.000	2.000	2.000
**RG mode**			
S50	3.000	3.000	3.000
S100	1.000	1.000	1.000
S275	1.000	1.000	1.000
S550	1.000	1.000	1.000
Letters	2.000	2.000	2.000
Coinflip	1.000	1.000	1.000
**Phi 2**			
S50	−1.753	−1.753	−1.753
S100	0.755	0.755	0.755
S275	0.009	0.009	0.009
S550	−0.302	−0.302	−0.302
Letters	−0.195	−0.195	−0.195
Coinflip	−3.676	−3.676	−3.676
**Phi 3**			
S50	−3.008	−3.008	−3.008
S100	0.905	0.905	0.905
S275	−0.676	−0.676	−0.676
S550	−0.985	−0.985	−0.985
Letters	0.506	0.506	0.506
Coinflip	−5.971	−5.971	−5.971
**Phi 4**			
S50	−0.312	−0.312	−0.312
S100	−0.374	−0.374	−0.374
S275	−0.845	−0.845	−0.845
S550	−0.816	−0.816	−0.816
Letters	−0.195	−0.195	−0.195
Coinflip	0.024	0.023	0.023
**Phi 5**			
S50	−1.336	−1.336	−1.336
S100	−0.531	−0.531	−0.531
S275	−0.662	−0.662	−0.662
S550	−0.226	−0.226	−0.226
Letters	0.183	0.183	0.183
Coinflip	13.384	13.384	13.384
**Phi 6**			
S50	−0.534	−0.534	−0.534
S100	−0.562	−0.562	−0.562
S275	0.789	0.789	0.789
S550	−0.528	−0.528	−0.528
Letters	−0.173	−0.173	−0.173
Coinflip	−3.307	−3.307	−3.307
**Phi 7**			
S50	−3.544	−3.544	−3.544
S100	0.355	0.355	0.355
S275	0.059	0.059	0.059
S550	−0.061	−0.061	−0.061
Letters	−0.466	−0.466	−0.466
Coinflip	−4.317	−4.317	−4.317

*Differences in values (except RNG and RNG2 of the letter sequence) between RGcalc and RandseqR (classical) values are attributed to the higher precision of R*.

Table 1 shows the output for all six sequences using *RGcalc* and *RandseqR* (with outcome values expressed in the classic and default mode). Minor differences occur between *RGcalc* output and *RandseqR* output, which can be attributed to the higher precision of R compared to 1998 Visual Basic. The rather large difference between *RGcalc* and *RandseqR* for **RNG** and **RNG2** in the letter sequence, however, cannot be attributed to this difference. The origin of this difference is unclear, but it is possible the *RGcalc* code is not optimized for sequences with more than nine response alternatives (like letter sequences). One of the major upsides of *RandseqR* over *RGcalc* is that *RandseqR* enables processing of a multitude of sequences without manually handling the in- and output. Furthermore, *RandseqR* accepts all types of input (e.g., coinflips), as long as the response alternatives are defined. In contrast, *RGcalc* does not accept string variables unless heads and tails are manually converted to digits. The one exception is that *RGcalc* does accept letters as input.

Table 2 shows the RQA output for each sequence. The following line of code was used to calculate RQA measures:

$$crqa\text{\_}cl\left(x,\;emRad=0.1\;doPlot=“rp^{\prime \prime }\right),$$

were x is one of the six sequences. For a full explanation on *crqa_cl* see the documentation in casnet. The only term in *crqa_cl* that deviates from the default setting is radius (emRad), which is set to a value lower than 1. The term *doPlot* creates the RPs for the sequences, which are showed in Figure 1.

**Table 2:** RQA output.

**Sequence**	**RR**	**DET**	**Lmax**	**L**	**Lentr**	**LAM**	**Vmax**	**TT**	**Ventr**
S50	0.106	0.211	4.000	2.333	0.721	0.128	2.000	2.000	0.000
S100	0.112	0.198	4.000	2.135	0.410	0.259	4.000	2.221	0.532
S275	0.109	0.205	5.000	2.112	0.362	0.214	3.000	2.114	0.354
S550	0.111	0.213	6.000	2.147	0.438	0.200	3.000	2.122	0.372
Letters	0.039	0.077	3.000	2.055	0.212	0.055	3.000	2.078	0.274
Coinflip	0.491	0.734	10.000	2.902	1.306	0.735	5.000	2.721	1.097

Taking a closer look at the RQA output, all four number sequences are more or less stable, as is to be expected for randomly generated number sequences. Instead, many of the randomization measures (Table 1) show a clear increasing or decreasing trend with increasing *N*. The most obvious in this regard is **NSQ**, which shows a downward trend and eventually becomes 0 when all possible digram pairs are used. Other measures that show a downward or upward trend are **R**, **RNG**, **RNG2**, **TPI**, and **Phi**. These measures project ratios between the observed number or digram distributions and the expected number or digram distribution. By increasing *N* (i.e., the amount of numbers in the sequence) in random sequences, the observed distribution better approximates the expected distribution.

Conceptually **RR** is somewhat similar to **R** as the amount of recurring numbers increases with increasing **redundancy**. The calculation differs, however, because **redundancy** is proportioned to the maximum number of black dots possible, instead of the number of white dots. Furthermore, the **phi-indices** deal with recurring numbers at a certain time lag and are, therefore, somewhat related to **RR**. **RR**, however, is a measure of recurring numbers at all time lags. **phi 2** is related to **LAM**, since a recurring number at time lag 1 (i.e., **phi 2**) is considered a vertical line structure and therefore counts toward **LAM**. For most other randomization measures, the calculation uses diagrams (i.e., a combination of two digits), instead of recurring numbers and, therefore, have no obvious equivalence with RQA. Nonetheless, certain diagrams might reoccur, like sequences as measured by **RNG** and **adjacency**, and therefore counts toward **DET**, but only for sequences at time lag 1. If the average line length is close to 2 (time lag 1), measure like **RNG** are closer related to **DET** than when the average line length increases. Earlier research using principal component analysis supports these conceptual similarities between randomization and RQA measures (Oomens et al., 2015).

## Data Availability Statement

The original contributions presented in the study are included in the article/Supplementary Material, further inquiries can be directed to the corresponding author.

## Author Contributions

WO, FH, JM, and JE designed and planned the study. WO and FH wrote the R code for randseqR. WO drafted the manuscript. FH, JM, and JE revised and critically reviewed the manuscript for important content. All authors read and authorized the final version of the manuscript.

## Conflict of Interest

The authors declare that the research was conducted in the absence of any commercial or financial relationships that could be construed as a potential conflict of interest.

## Supplementary Material

The *RandseqR* package is available at: https://github.com/FredHasselman/randseqR. To download and install *RandseqR* into your R library use the following line of code:

$$remotes::install\text{\_}github\left(“FredHasselman/randseqR^{\prime \prime }\right).$$

The *RGcalc* software is available at: https://www.lancaster.ac.uk/staff/towse/rgcpage.html.
