# Supplementary material for: RandseqR: An R Package for Describing Performance on the Random Number Generation Task
Source: Front Psychol. 2021 May 4;12:629012. doi: 10.3389/fpsyg.2021.629012 (PMC8129161; doi:10.3389/fpsyg.2021.629012)
Supplement: Supplementary file 1 [file Data_Sheet_1.pdf]

# RandseqR: The calculation of Phi Index

Wouter Oomens[1,2], Joseph H.R. Maes[2], Fred Hasselman[3], & Jos I.M. Egger[1,2]

[1] Centre of Excellence for Neuropsychiatry, Vincent van Gogh Institute for Psychiatry, Venray, Netherlands

[2] Donders Institute for Brain, Cognition and Behavior, Radboud University, Nijmegen, Netherlands

[3] School for Pedagogical and Educational Science, Radboud University, Nijmegen, Netherlands

[4] Stevig, Specialized and Forensic Care for People with Intellectual Disabilities, Dichterbij, Oostrum, The Netherlands

## Author Note

Correspondence concerning this article should be addressed to Wouter Oomens, Centre of Excellence for Neuropsychiatry, Vincent van Gogh Institute for Psychiatry, Stationsweg 46, Venray 5803 AC, Netherlands. E-mail: w.oomens@donders.ru.nl

RandseqR: The calculation of Phi Index

## The calculation of Phi Index

The calculation of **Phi** is an iterating process in which each *phi order* is computed consecutively. During each iteration the values of each cell in table 1 are determined, whereas the expected repeats and alternates depend on the number of observed repeats and alternates of the previous iteration (i.e., *phi order* $-1$)[1].

The **Phi indices** are the outcome of a chi-square analysis on the cells of table 1 using the formula:

$$Phi = 100 * \sqrt{(\frac{Chi}{n * a})},$$

where $n$ is the length of the number sequence and $a$ is the number of response alternatives. In the case that the number of observed alternatives ($b$) is greater than the number of expected alternatives ($d$), Phi is multiplied by $-1$. Therefore, possible Phi results range from $-\inf$ to $+\inf$, where negative Phi equals less number repeats than expected. In the formula for Phi, $Chi$ is determined as follows: $Chi = B1 + B2 + B3 + B4$, where

$$B1 = \frac{(a - \frac{A*C}{grand\ Total})^2}{\frac{A*C}{grand\ Total}},$$

$$B2 = \frac{(b - \frac{A*D}{grand\ Total})^2}{\frac{A*D}{grand\ Total}},$$

$$B3 = \frac{(c - \frac{B*C}{grand\ Total})^2}{\frac{B*C}{grand\ Total}},$$

$$B4 = \frac{(d - \frac{B*D}{grand\ Total})^2}{\frac{B*D}{grand\ Total}}.$$

To determine the number of observed repeats and alternates, the time series is converted to a binary series of zeroes and ones for each response alternative. For each of

[1] For **phi 2** the *phi order* $-1$ is either the observed frequency or inverse frequency of each response alternative.

these binary series the sum of all repeating engrams at time lag $x$ is the number of observed repeats. The sum of all alternating engrams at time lag $x$ is the number of observed alternates. An engram is considered a repeat if the first and last digit of that engram are the same[2]. the **phi 2** engrams are: 00, 01, 10, and 11, of which 00 and 11 are considered repetitions. For **phi 3** there are eight engrams (i.e., 000, 001, 010, 011, 100, 101, 110, and 111), of which 000, 010, 101, and 111 are considered repetitions. **phi 4** has sixteen engrams and so forth.

Next, the amount of expected repetitions and alternations are determined for each response alternative. For each of the binary series, the expected value is the product of the frequency of observed engram combination[3] in the binary series at *Phi order* $-1$ divided by a value $M$. The value $M$ depends on the *phi order*: for **phi 2** $M$ is equal to $n$, for **phi 3** $M$ is the (inverse) observed frequency of the response alternative, and for all other **phi grams** $M$ is equal to the observed frequency of the binary engram of length *phi* $-2$ (the middle section). The sum of all repeating engrams at time lag $x$ is the amount of expected repeats, while the sum of all alternating engrams at time lag $x$ is the amount of expected alternates.

To give an example, consider the following time series of $n = 10$ and $a = 3$ response alternatives: **1311213321**. For each response alternative the times series is converted to a binary series:

$$response\ alternative\ 1:\ 1011010001,$$

$$response\ alternative\ 2:\ 0000100010,$$

$$response\ alternative\ 3:\ 0100001100.$$

[2] note that both true repetitions, a repetition of the target response at time lag $x$, and pseudo repetitions, a repetition of two non-target responses at time lag $x$, are considered observed repetitions.

[3] for an engram of length *phi*, the observed frequency of the left section of length *phi* $-1$ of that engram is multiplied by the observed frequency of the right section of length *phi* $-1$ of that engram. For example, to determine the expected value for the engram 101 the observed frequency for the engram 10 is multiplied by the observed frequency for the engram 01.

For **phi 2** the amount of repetitions (either 00 or 11) in our example is: 3, 5, and 5, respectively for a total of 13 observed repeats. The amount of alternations (either 01 or 10) in our example is: 6, 4, and 4, respectively for a total of 14 observed alternates (see table 2).

For the expected repetition 00 the *inverse frequency* (the frequency of a response alternative **not** occurring in the time series) is multiplied by itself and divided by 10 for each response alternative, which gives the values: 2.5, 6.4, and 4.9, respectively. For the expected repetition 11 the *observed frequency* is multiplied by itself and divided by 10 for each response alternative, which gives the values: 2.5, 0.4, and 0.9, respectively. The expected repeats is the sum of these six values for a total of 17.6 expected repeats. For the expected alternation 01 the inverse frequency is multiplied by the observed frequency and divided by 10 for each response alternative, which gives the values: 2.5, 1.6, and 2.1 respectively. For the expected alternations 10 the observed frequency is multiplied by the inverse frequency and divided by 10 for each response alternative, which is the same as the expected alternation 01. The expected alternates is the sum of these six values for a total of 12.4 expected alternates (see table 2).

By applying the formulas B1 to B4 to the values of table 2 gives a Chi value of 0.6323 and a Phi value of 14.5178. Since the number of observed alternatives is greater than the number of expected alternatives this result is multiplied by $-1$, for a *Phi 2* value of $-14.5178$.

Table 1

*Table 1: observed/expected table for the calculation of phi*

| Variable | Repeats | Alternates | Row total |
|---|---|---|---|
| Observed | a | b | A |
| Expected | c | d | B |
| Column total | C | D | Grand total |

Table 2

*Table 2: example of the observed/expected table for the calculation of phi*

| Variable | Repeats | Alternates | Row total |
|---|---|---|---|
| Observed | 13.00 | 14.00 | 27.00 |
| Expected | 17.60 | 12.40 | 30.00 |
| Column total | 30.60 | 26.40 | 57.00 |
